# Identification of Licopyranocoumarin and Glycyrurol from Herbal Medicines as Neuroprotective Compounds for Parkinson's Disease

**DOI:** 10.1371/journal.pone.0100395

**Published:** 2014-06-24

**Authors:** Takahiro Fujimaki, Shinji Saiki, Etsu Tashiro, Daisuke Yamada, Mitsuhiro Kitagawa, Nobutaka Hattori, Masaya Imoto

**Affiliations:** 1 Department of Biosciences and Informatics, Faculty of Science and Technology, Keio University, Yokohama, Japan; 2 Department of Neurology, Juntendo University School of Medicine, Bunkyo, Tokyo; Hokkaido University, Japan

## Abstract

In the course of screening for the anti-Parkinsonian drugs from a library of traditional herbal medicines, we found that the extracts of *choi-joki-to* and *daio-kanzo-to* protected cells from MPP^+^-induced cell death. Because *choi-joki-to* and *daio-kanzo-to* commonly contain the genus *Glycyrrhiza*, we isolated licopyranocoumarin (LPC) and glycyrurol (GCR) as potent neuroprotective principals from *Glycyrrhiza*. LPC and GCR markedly blocked MPP^+^-induced neuronal PC12D cell death and disappearance of mitochondrial membrane potential, which were mediated by JNK. LPC and GCR inhibited MPP^+^-induced JNK activation through the suppression of reactive oxygen species (ROS) generation, thereby inhibiting MPP^+^-induced neuronal PC12D cell death. These results indicated that LPC and GCR derived from *choi-joki-to* and *daio-kanzo-to* would be promising drug leads for PD treatment in the future.

## Introduction

Parkinson's disease (PD) is a common neurodegenerative disease characterized by progressive dopaminergic neuronal cell death in the substantia nigra par compacta of the midbrain. The main symptoms of PD are movement disorders such as tremors, bradykinesia/akinesia, rigidity, postural instability, and gait abnormalities. Although deep-brain stimulation and oral administration of L-dopa, dopamine agonists and amantadine hydrochloride have been well established as symptomatic treatments, there are no therapies to completely cure patients with the disorder [1]. Mitochondrial dysfunction, especially dysfunction of the mitochondrial electron transport chain mainly relying on complex I activity, has been implicated in the disease's pathogenesis. In addition to defects of complex I in postmortem brains, skeletal muscle and platelets of patients with PD [2], [3], [4], [5], [6], cybrid cells containing mtDNA derived from PD platelets have indicated complex I defects [7], [8], [9]. Because various rodents treated with mitochondrial toxins such as rotenone, 1-methyl-4-phenyl-1,2,3,6-tetrahydropyridine (MPTP), and its toxic metabolite 1-methyl-4-phenylpyridinium (MPP^+^) show motor deficits associated with selective loss of dopaminergic neurons, they have been widely used as acquired PD models [10], [11], [12], [13], [14], [15]. Selegiline, a medication widely used at present, has the capacity to protect dopamine neurons by inhibiting MAO-B oxidation for conversion of MPTP into MPP^+^ and blocking the formation of free radicals derived from the oxidative metabolism of dopamine [16], [17]. Also, MPP^+^ models offer unexploited therapeutic potential for some atypical antipsychotics (olanzapine, aripiprazole, and ziprasidone) and the anticonvulsant zonisamide in PD, and new mechanisms of neuroprotective effects of FLZ (which activates HSP27/HSP70) and paeoniflorin (which modulates autophagy) have led to treatments for PD [18], [19], [20], [21].

Herbal medicines are employed to treat PD in ancient medical systems in Asian countries such as India, China, Japan, and Korea based on anecdotal and experience-based theories [22]. The traditional herbal medicines *yi-gan san* and *modified yeoldahanso-tang* have neuroprotective effects and can rescue dopaminergic neurons from MPP^+^/MPTP toxicity using both *in vitro* and *in vivo* methods [23], [24]. Several compounds derived from herbal medicines also exert anti-Parkinsonian activities. For instance, ginsenoside Rb1 isolated from *Panax ginseng C. A. Meyer*, 3-*O*-demethylswertipunicoside isolated from *S. punicea*, and salidroside isolated from *Rhodiola rosea L*., have been reported to attenuate MPP^+^-induced neurotoxicity in PC12 cells *in vitro* [25], [26], [27]. However, clinical evidence for the efficacy and safety of these herbal medicines for PD is insufficient [28]. Therefore, in this study, we screened a library containing 128 traditional herbal medicines, which have been used clinically for at least 10 years in Japan, focusing on their neuroprotective effects using PD-like cellular models of cell death by mitochondrial toxins, and found the anti-Parkinsonian herbal medicines *choi-joki-to* and *daio-kanzo-to*. Moreover, we identified licopyranocoumarin and glycyrurol derived from the genus *Glycyrrhiza* as common components contained in these two herbal medicines, and found they exerted neuroprotective effects against MPP^+^-induced toxicity.

## Results

### Identification of *choi-joki-to* and *daio-kanzo-to* as potent neuroprotective herbal medicines using i*n vitro* PD-like model screening

Rotenone, a direct inhibitor of mitochondria complex I, is usually employed to mimic Parkinsonism *in vitro* and *in vivo* [29]. Treatment of NGF-differentiated PC12D cells [30] with 0.3 µM of rotenone for 48 h caused marked cell death as evaluated by the trypan blue dye exclusion assay. Using this PD-like model, we screened a library containing 128 traditional herbal medicines, which have been used clinically in Japan, focusing on preventive effects against rotenone-induced cell death of NGF-differentiated PC12D cells.

**Figure 1:**
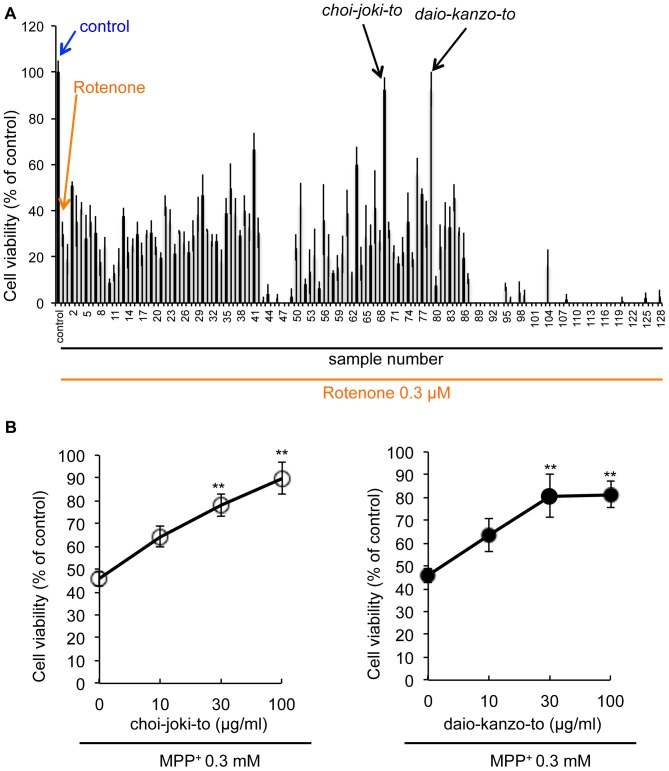
Two herbal medicines, *daio-kanzo-to* and *choi-joki-to*, identified as neuroprotective agents in the course of screening. (**A**) NGF-differentiated PC12D cells were treated with 0.3 µM rotenone and herbal medicine extract for 48 h. Cell viability was evaluated by trypan blue dye exclusion assay. (**B**) NGF-differentiated PC12D cells were treated with various concentrations of *choi-joki-to* or *daio-kanzo-to* in the presence of 0.3 mM MPP^+^ for 48 h. Cell viability was evaluated by trypan blue dye exclusion assay. Values are the means of triplicate samples; bars, s.d. ^**^ *p*<0.01 compared with MPP^+^ group cells.

As a result, several ethyl acetate (EtOAc) extracts of herbal medicines showed suppressive effects against rotenone-induced cell death generally, but two traditional herbal medicines, *choi-joki-to* and *daio-kanzo-to* exerted significant neuroprotective effects against rotenone-induced neurotoxicity (Figure 1A). Furthermore, the EtOAc extracts of *choi-joki-to* or *daio-kanzo-to* also conferred dose-dependent protection from neuronal cell death induced by MPP^+^, another well-known PD-like cellular model (Figure 1B).

### Licopyranocoumarin and glycyrurol isolated from *Glycyrrhiza* as potent neuroprotective compounds

**Figure 2:**
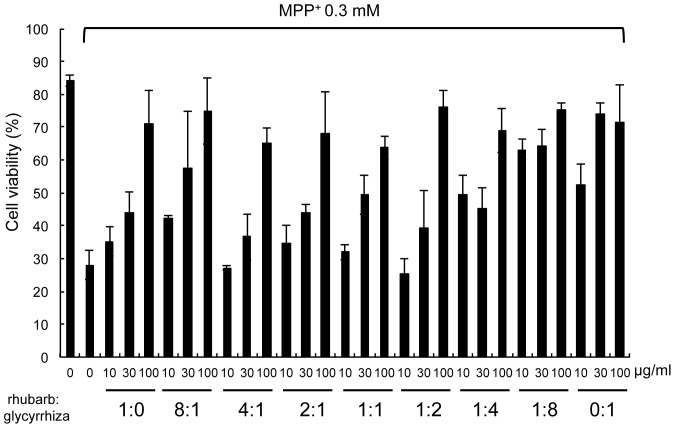
*Glycyrrhiza* prevented MPP^+^-induced cell death more potently than rhubarb. NGF-differentiated PC12D cells were treated with various concentrations of rhubarb and *Glycyrrhiza* (rhubarb:*Glycyrrhiza* ratio = 1∶0, 8∶1, 4∶1, 2∶1, 1∶1, 1∶2, 1∶4, 1∶8, 0∶1) in the presence of 0.3 mM MPP^+^ for 48 h. Cell viability was evaluated by trypan blue exclusion assay. Values are the means of three independent experiments; bars, s.d. ^**^ *p*<0.01 compared with MPP^+^ group cells.

**Figure 3:**
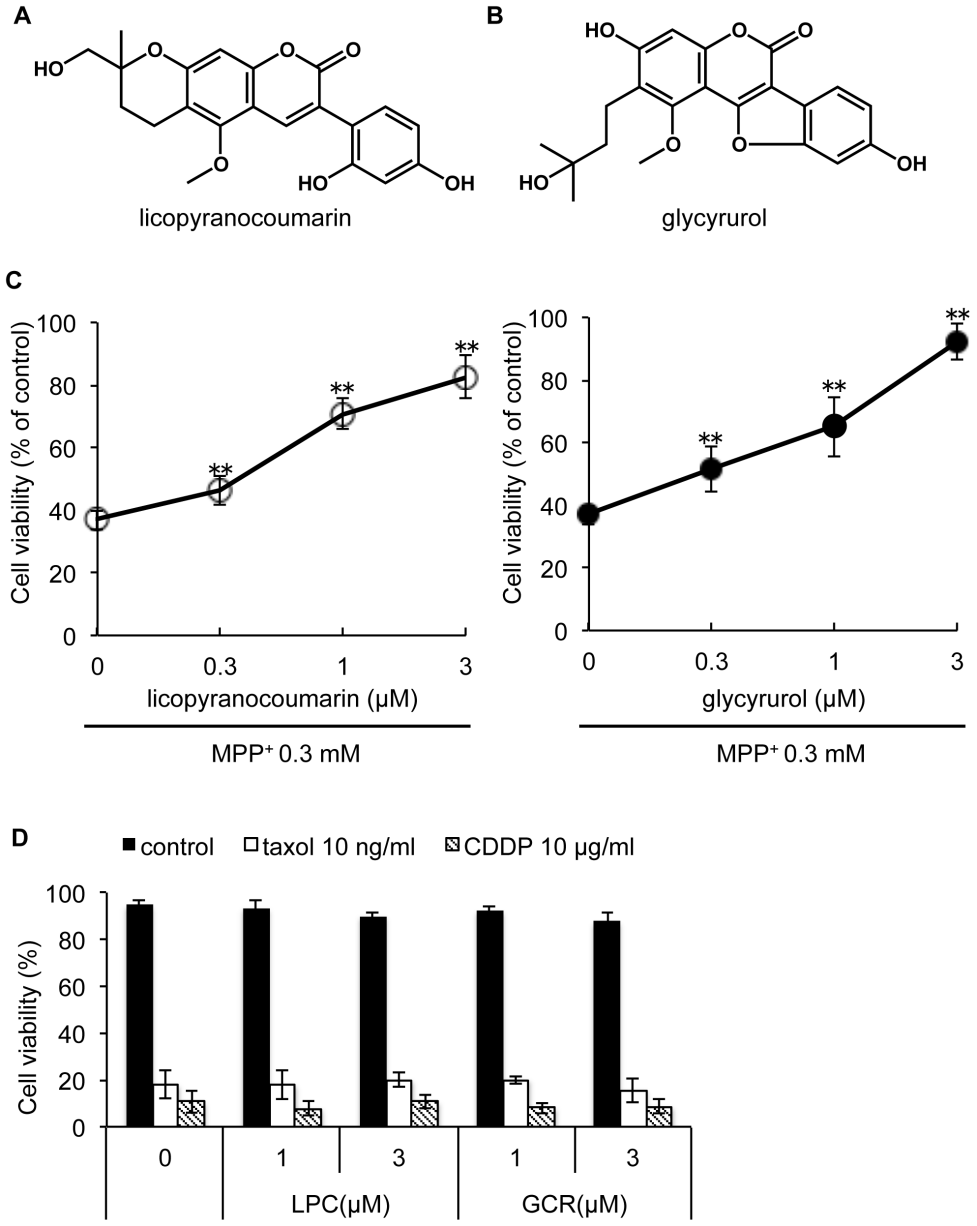
Licopyranocoumarin and glycyrurol prevented MPP^+^-induced cell death. Structures of (**A**) licopyranocoumarin (LPC) and (**B**) glycyrurol (GCR). (**C**) NGF-differentiated PC12D cells were treated with various concentrations of LPC or GCR in the presence of 0.3 mM MPP^+^ for 48 h. Cell viability was evaluated by trypan blue dye exclusion assay. (**D**) PC12D cells were treated with various concentration of LPC or GCR in the presence of 10 ng/ml taxol or 10 µg/ml cisplatin (CDDP) for 48 h. Values are the means of three independent experiments; bars, s.d. ^**^ *p*<0.01 compared with MPP^+^ group cells.

**Figure 4:**
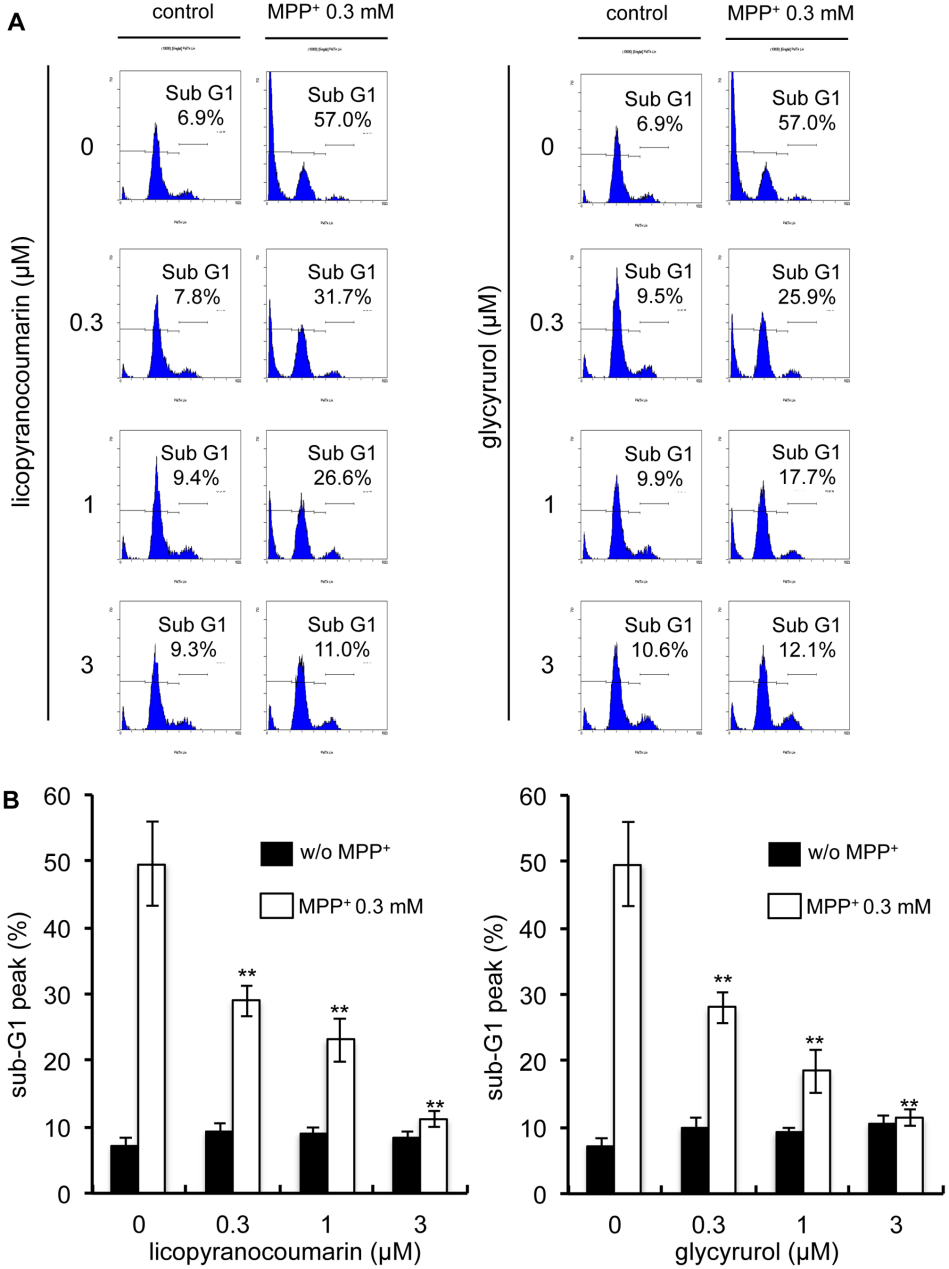
Licopyranocoumarin and glycyrurol attenuated MPP^+^-induced apoptosis. (**A**) NGF-differentiated PC12D cells were treated with various concentrations of licopyranocoumarin or glycyrurol in the presence of 0.3 mM MPP^+^ for 48 h. Collected cells were stained with PI and analyzed by flow cytometry. (**B**) The sub G1 ratio was analyzed. Values are the means of three independent experiments; bars, s.d. ^**^ *p*<0.01 compared with MPP^+^ group cells.

Next, we attempted to identify the major components responsible for neuroprotective effects contained in *choi-joki-to* and *daio-kanzo-to*. First, we noted that both *choi-joki-to* and *daio-kanzo-to* commonly contain rhubarb and *Glycyrrhiza* species, at the ratio of 2∶1 (Table 1). Therefore, we examined whether this 2∶1 ratio of rhubarb to *Glycyrrhiza* is important for neuroprotective effects against MPP^+^-induced toxicity. As shown in Figure 2, rhubarb and *Glycyrrhiza* contained in *choi-joki-to* and *daio-kanzo-to* at 2∶1 is not a special ratio necessary for neuroprotective effects, but rather increased *Glycyrrhiza* content potentiated the neuroprotective activity against MPP^+^-induced cell death. Thus, we attempted to isolate the active principle responsible for neuroprotective effects from EtOAc extract of *Glycyrrhiza* by monitoring the inhibitory activity of MPP^+^-induced NGF-differentiated PC12D cell death using a trypan blue dye exclusion assay. As a result, we isolated 10.8 mg of licopyranocoumarin (LPC) and 4.0 mg of glycyrurol (GCR) from 50 g of *Glycyrrhiza* powder as potent neuroprotective compounds (Figure 3A, B). Both LPC and GCR markedly blocked MPP^+^-induced cell death in a dose-dependent manner with IC_50_ values of 0.9 µM and 1.2 µM, respectively (Figure 3C). Furthermore, both LPC and GCR did not show cytoprotective effects against other toxins, such as taxol and cisplatin (CDDP) even at 3 µM concentration, which significantly suppressed MPP^+^-induced cell death in PC12D cells. Therefore, cytoprotective ability of LPC and GCR may specific for mitochondrial toxins (Figure 3D). To further verify the inhibitory effect of LPC and GCR on MPP^+^-induced cell death, PC12D cells were labeled with PI and histogram analysis-related nuclear DNA contents were ascertained by flow cytometry. By the treatment of PC12D cells with 0.3 mM of MPP^+^, NGF-differentiated PC12D cells with DNA content below G1 phase levels (defined as hypodiploid sub-G1 peak) were distinguishable in the population as compared with control levels (49.63±6.41% versus 7.23±1.04% of cells in sub-G1, respectively) (Figure 4A,B). LPC or GCR alone did not show any effects on the overall population of cells. However, they decreased the percentage of MPP^+^-induced cell death by 11.2–29.0% and 11.4–28.0% (values are the mean of average of three data), respectively (Figure 4A,B), confirming that LPC and GCR inhibited MPP^+^-induced cell death.

**Table 1:** Crude drugs constituents of “*choi-joki-to*” and “*daio-kanzo-to*”.

*choi-joki-to*		*daio-kanzo-to*	
Scientific names	Contents (g)	Scientific names	Contents (g)
rhubarb	2	rhubarb	4
*glycyrrhiza*	1	*glycyrrhiza*	2
Salt cake	0.5		

### Licopyranocoumarin and glycyrurol attenuate the MPP^+^-induced decrease in mitochondrial membrane potential

**Figure 5:**
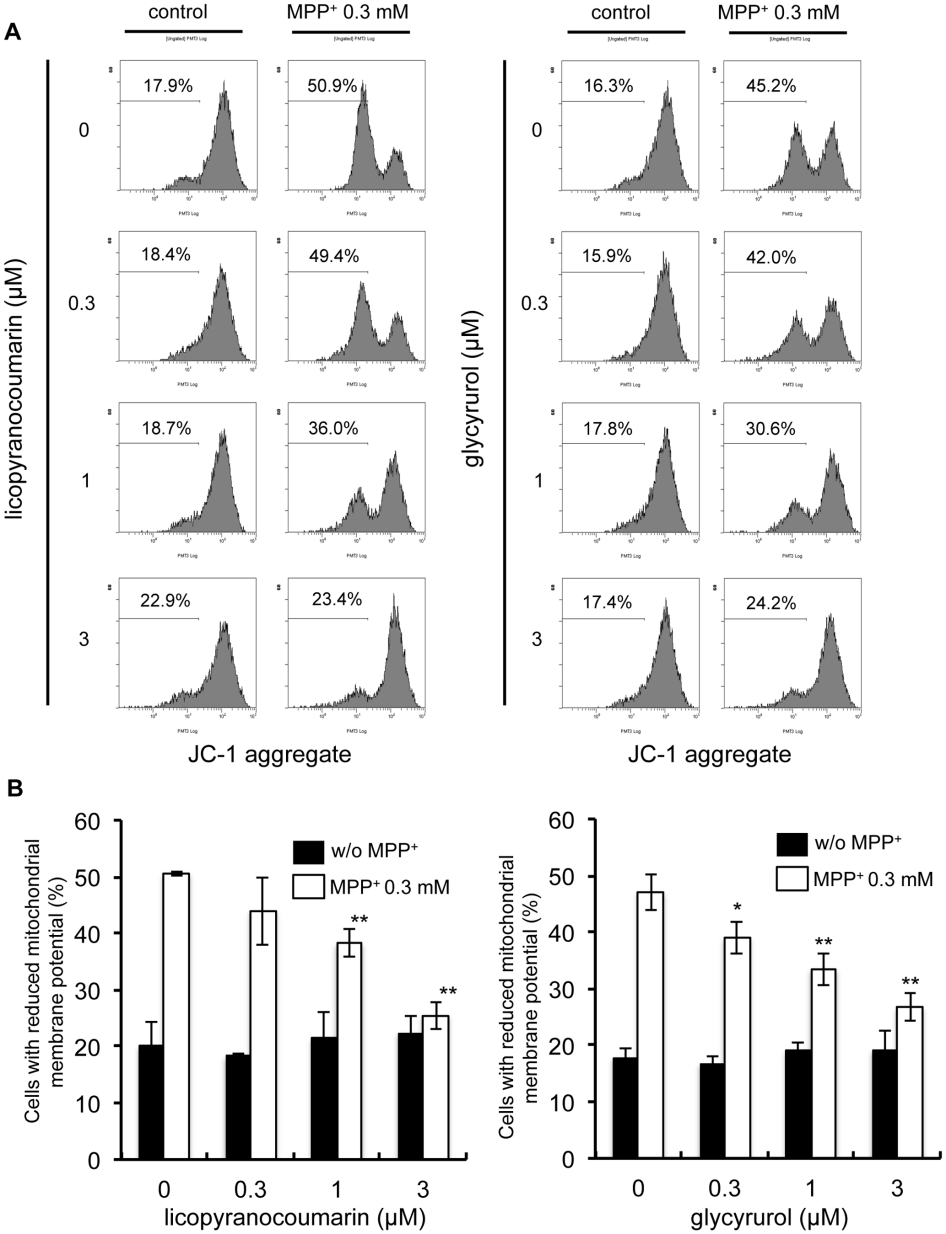
Licopyranocoumarin and glycyrurol protected cells against MPP^+^-induced disappearance of mitochondrial membrane potential. (**A**) NGF-differentiated PC12D cells were treated with various concentrations of licopyranocoumarin or glycyrurol in the presence of 0.3 mM MPP^+^ for 48 h. Collected cells were stained with JC-1 and analyzed by flow cytometry. (**B**) The ratio of cells exhibiting disappearance of mitochondrial membrane potential was analyzed. Values are the means of three independent experiments; bars, s.d. ^*^ *p*<0.05, ^**^ *p*<0.01 compared with MPP^+^ group cells.

MPP^+^ is a well-known inhibitor of mitochondria complex I and induces mitochondrial dysfunction. Because LPC or GCR suppressed MPP^+^-induced cell death, we next surveyed the effect of LPC and GCR on MPP^+^-mediated loss of mitochondrial membrane potential (ΔΨ_mit_) using JC-1 dyes. As shown in Figure 5, by the treatment of PC12D cells with 0.3 mM of MPP^+^ for 48 h, ΔΨ_mit_ was decreased to 45–50% as estimated from decrease of JC-1 aggregate fluorescence. LPC or GCR alone did not affect ΔΨ_mit_. Compared with the group treated with MPP^+^ alone, fluorescent intensities increased in a dose-dependent manner following addition of LPC and GCR individually, indicating that LPC and GCR each inhibited MPP^+^-induced decrease of ΔΨ_mit_.

### Licopyranocoumarin and glycyrurol counteract MPP^+^-induced ROS production

**Figure 6:**
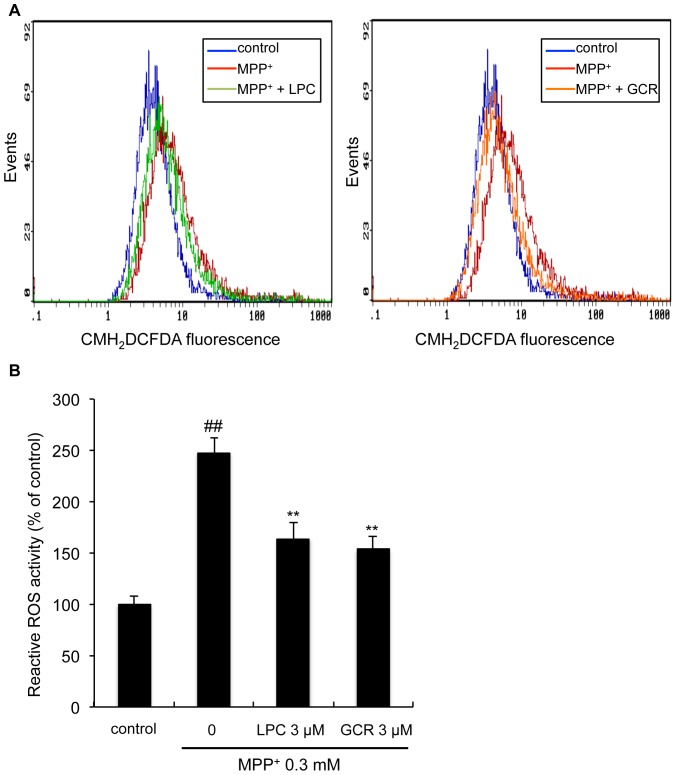
Licopyranocoumarin and glycyrurol decreased MPP^+^-induced intracellular ROS generation. (**A**) NGF-differentiated PC12D cells were pre-incubated for 1 h with 3 µM licopyranocoumarin (LPC) or 3 µM glycyrurol (GCR), then treated with 0.3 mM MPP^+^ for 12 h. Then, the samples were loaded with 2.5 µM CM-H_2_DCFDA and the fluorescence intensities were measured by flow cytometry. (**B**) The ratio of cells exhibiting ROS production was analyzed. Values are the means of four independent experiments; bars, s.d. ^##^ *p*<0.01 compared with control cells. ^**^ *p*<0.01 compared with MPP^+^ group cells.

MPP^+^ has been extensively reported to evoke generation of reactive oxygen species (ROS). Figure 6 showed cytofluorometric histograms of NGF-differentiated PC12D cells after 12 h of treatment with 0.3 mM MPP^+^ upon staining with CMH_2_DCFDA. ROS levels were significantly increased from 100±7.8% (control level) to 247±14.9% (p<0.001). However, the generation of intracellular ROS was reduced to 164±15.7% (p<0.01) and 153±13.0% (p<0.01) by the addition of 3 µM LPC and 3 µM GCR, respectively.

### Antioxidant activities of licopyranocoumarin and glycyrurol *in vitro*

**Figure 7:**
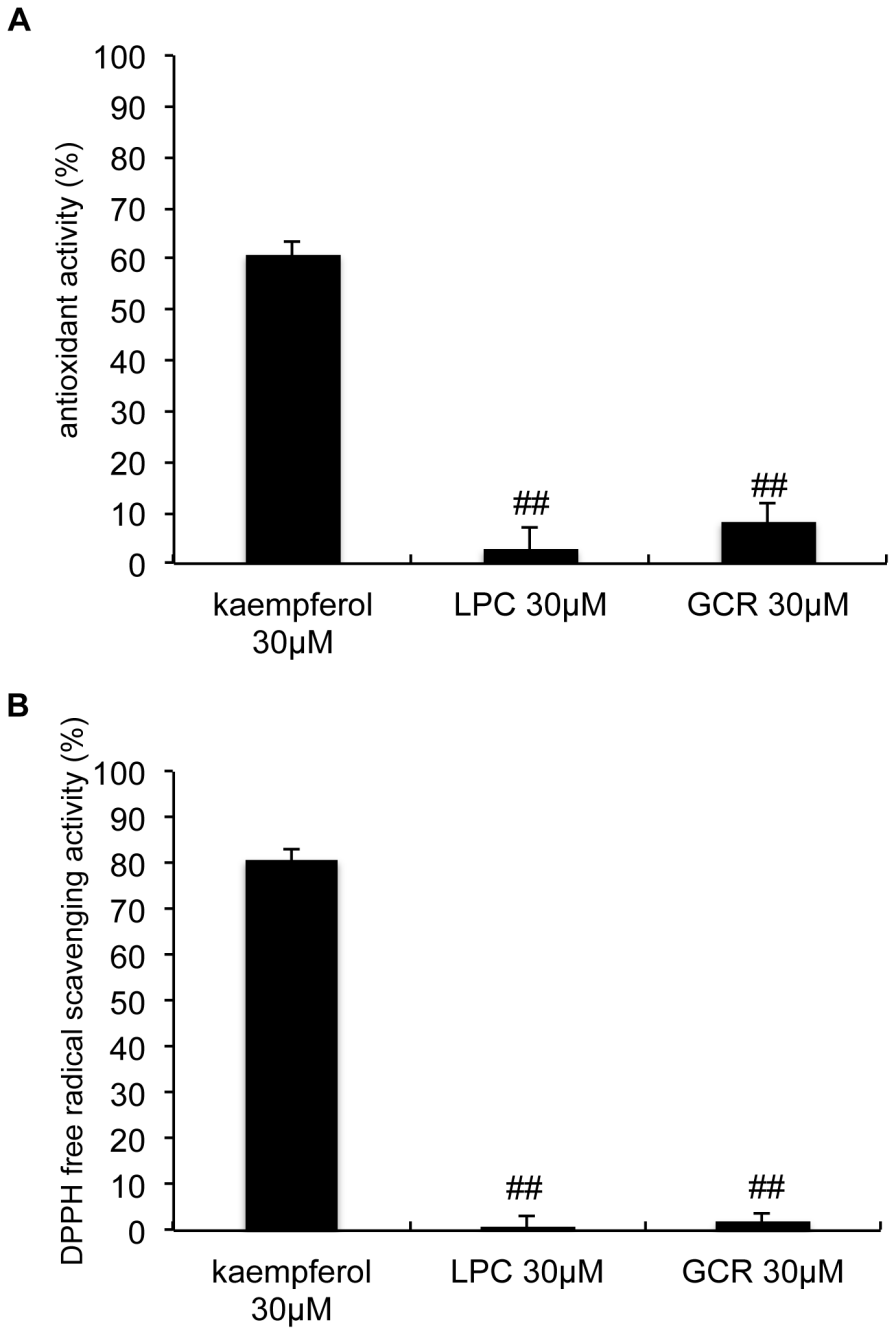
Licopyranocoumarin and glycyrurol lacked potency for scavenging free radicals. Antioxidant activities of licopyranocoumarin (LPC) and glycyrurol (GCR) were measured by (**A**) a β-carotene bleaching assay system and (**B**) a DPPH radical scavenging assay. Kaempferol served as the positive control. Values are the means of three independent experiments; bars, s.d. ^##^ *p*<0.01 compared with antioxidant activity of kaempferol.

Because treatment of PC12D cells with LPC and GCR each effectively reduced MPP^+^-induced ROS generation, the free radical scavenging activities of these two compounds were examined. When the antioxidant activity of LPC and GCR were evaluated by β-carotene bleaching assay, LPC and GCR inhibited less than 10% of the carotene bleaching even at the final concentration of 30 µM (Figure 7A). The DPPH free radical scavenging potentials of LPC and GCR at 30 µM each showed little to no scavenging activity (Figure 7B). These results indicated that LPC and GCR did not possess antioxidant activity *in vitro*.

### Licopyranocoumarin and glycyrurol attenuate JNK activity induced by MPP^+^

**Figure 8:**
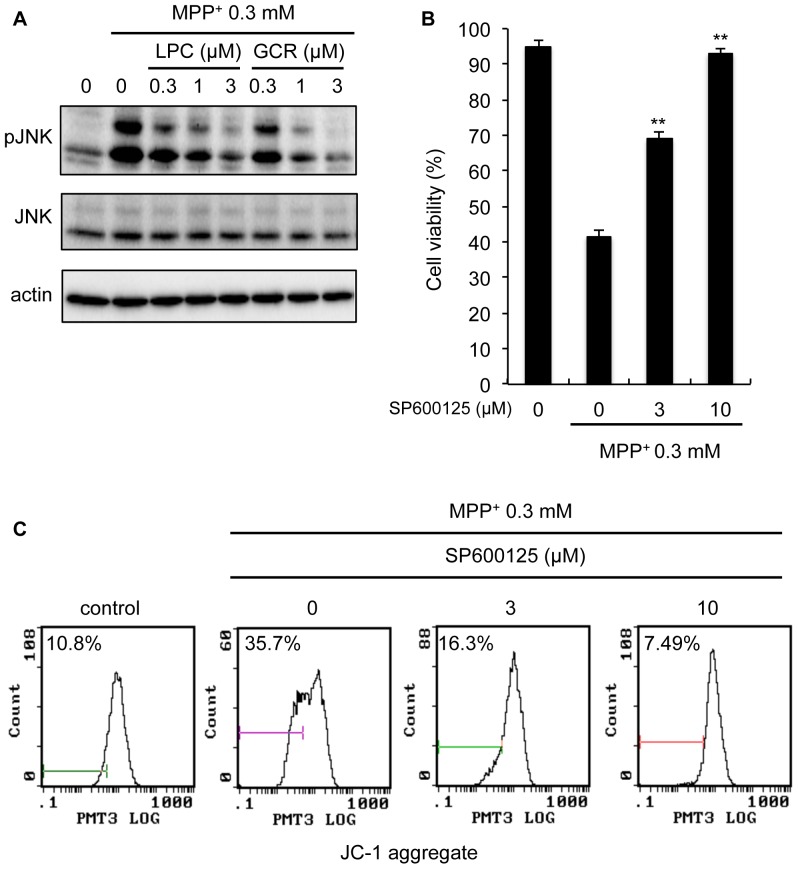
Licopyranocoumarin and glycyrurol attenuated MPP^+^-induced JNK activation. (**A**) NGF-differentiated PC12D cells were treated with various concentrations of licopyranocoumarin (LPC) or glycyrurol (GCR) and 0.3 mM MPP^+^ for 36 h, and JNK and phosphor-JNK level were detected by Western blot. NGF-differentiated PC12D cells were treated with SP600125 and 0.3 mM MPP^+^ for 48 h. Thereafter (**B**) cell viability was measured by trypan blue dye exclusion assay and (**C**) mitochondrial membrane potentials were assessed by JC-1 assay. Values of (B) are the means of three independent experiments; ^**^ *p*<0.01 compared with MPP^+^ group cells.

It is well-established that JNK plays a central role in the mediation of MPP^+^-induced neurotoxicity [31], [32], [33], [34]. Particularly, MPP^+^-induced ROS generation is reported to be closely associated with JNK activation [35]. Thus, we investigated whether the ability of LPC or GCR to reduce MPP^+^-induced cell death involves the alteration of JNK signaling in MPP^+^-induced neurotoxicity. As shown in Figure 8A, phosphorylated JNK levels were increased after exposure to MPP^+^ for 36 h, and treatment with LPC or GCR significantly reduced the expression levels of the phosphorylated protein. In addition, a JNK inhibitor, SP600125, led to attenuation of the MPP^+^-induced neuronal cell death and decreased ΔΨ_mit_ (Figure 8B, C). These results suggest that MPP^+^-induced lowering of ΔΨ_mit_, which leads to neuronal cell death, were mediated by JNK, and neuroprotective activity of LPC and GCR against MPP^+^-induced neuronal cell death might be due to downregulation of ROS generation, resulting in the inhibition of JNK activation.

## Discussion

Both *choi-joki-to* and *daio-kanzo-to* are traditional herbal medicines available in Japan (called *kanpo* in Japan in particular) that are usually used for laxative products. In the laboratory, *choi-joki-to* exhibited oxygen radical scavenging capacity [36] and inhibited the progression of atheroma in a KHC rabbit model [37], On the other hand, *daio-kanzo-to* has provided inhibition of amylase activity in mouse plasma and gastrointestinal tube [38], inhibition of cholera toxin [39], and inhibitory effects on drug oxidations [40]. In this study, we have demonstrated that *choi-joki-to* and *daio-kanzo-to* had neuroprotective effects against MPP^+^- and rotenone-induced toxicity in NGF-differentiated neuronal PC12D cells. Furthermore, we identified that *Glycyrrhiza*, commonly contained in these two herbal medicines, possessed potent neuroprotective activity against MPP^+^-induced toxicity. *Glycyrrhiza* is contained in a number of traditional herbal medicines including *yi-gan san* previously identified as neuroprotective agents against mitochondrial toxins, therefore, we investigated relationships between the neuroprotective effects of traditional herbal medicines and their contents of *Glycyrrhiza*. The correlation coefficient between neuroprotective effects of traditional herbal medicines and contents of *Glycyrrhiza* in each herbal medicine was calculated at 0.20 (Figure S1), indicating a very weak relationship. This weak relationship might be explained by our finding that higher concentration of *Glycyrrhiza* (300 µg/ml) showed cytotoxic effect in PC12D cells (Figure S2). Another possible explanation is that other constituent of traditional herbal medicines, such as rhubarb, also exerted neuroprotective effects in PC12D cells (Figure 2). Major components of *Glycyrrhiza* are triterpenoid saponins, and glycyrrhizin and its metabolite. These compounds show several potential health effects including anti-inflammatory, anti-viral, hepatoprotective, anti-cancer and immunomodulatory effects [41]. Therefore, at first we predicted that glycyrrhizin might be an active principle contained in *Glycyrrhiza* that suppressed MPP^+^- and rotenone-induced toxicity, but glycyrrhizin did not show such activities. Instead, we isolated the coumarin derivatives, licopyranocoumarin (LPC) and glycyrurol (GCR), as the most potent neuroprotective compounds in *Glycyrrhiza*. LPC isolated from *Glycyrrhiza* sp. has been reported to show several bioactivities, including anti-HIV effects and inhibition of CYP3A4 and the aryl hydrocarbon receptor antagonist [42], [43], [44]. On the other hand, GCR, which was very recently isolated from *Glycyrrhiza uralensis*, shows antithrombotic effects [45]. However, so far the neuroprotective effects of these two compounds have not yet been reported. This study has indeed revealed, for the first time, the potent neuroprotective activity of LPC and GCR in a PD-like cellular model system. LPC and GCR also inhibited rotenone-induced cell death in HeLa cells; however, the effects in HeLa cells were quite weak when compared to that seen in PC12D cells (Figure S3). Therefore, LPC and GCR seem to prefer to exert cytoprotection in neuronal cells. Oxidative stress associated with a general dysfunction of mitochondrial homeostasis is a leading hypothesis as a potential mechanism for dopaminergic neuronal degeneration in PD [46]. Postmortem analyses of the substantia nigra from PD patients confirm several oxidative stress-related alterations [47], [48], [49], and several toxins (rotenone, paraquat, and MPP^+^) used to produce PD-animal models directly and/or indirectly inhibit mitochondrial function, induce the production of ROS, and promote oxidative damage. Therefore, antioxidant ingredients are considered to be promising approach to prevent the disease progression. For example, α-tocopherol, coenzyme Q_10_ and catechols have been reported to exert neuroprotective effects by attenuating rotenone-induced oxidative stress on rotenone models *in vitro* and *in vivo* [50], [51], [52]. Likewise, we found that LPC and GCR attenuated the MPP^+^-induced increase in intracellular ROS generation (Figure 6A), indicating that inhibition of MPP^+^-mediated ROS generation is closely related to the neuroprotective effects of LPC and GCR. Several lines of evidence have suggested that ROS generation induces the activation of JNK signaling, and JNK represents one of the major signaling pathways implicated in PD pathogenesis. JNK activity is increased in MPTP animal models [53], [54], [55], [56], MPP^+^-treated cell culture models [35], [54], and rotenone neurotoxicity [57], [58]. Moreover, ROS-mediated activation of JNK almost inevitably leads to cell death. Indeed, we also confirmed that a JNK inhibitor, SP600125, suppressed MPP^+^-induced cell death (Figure 8B), and MPP^+^-induced activation of JNK and cell death were found to be inhibited by LPC and GCR under conditions where LPC or GCR inhibited the MPP^+^-mediated ROS generation (Figure 8A). Although the potential mechanisms by which JNK participates in MPP^+^-induced cell death remains to be fully determined, activation of JNK has been reported to mediate cell death by participating in the induction of mitochondrial permeability transition (mPT) and decrease of ΔΨ_mit_ in subsets of cell types [59], [60]. Because in our assay system SP600125 inhibited both cell death and the decrease in ΔΨ_mit_ induced by MPP^+^ (Figure. 8B and C), we consider the inhibition of the decrease in MPP^+^-induced ΔΨ_mit_ caused by LPC and GCR (Figure 5) to be due to the inhibition of ROS-mediated JNK activation.

Several neuroprotective compounds have significant antioxidant and free radical-scavenging activities. LPC and GCR are members of the coumarin compound family. There have been several reports on the antioxidant activities of coumarins [61], [62], [63], and LPC and GCR each inhibited MPP^+^-induced ROS generation. Nevertheless, neither LPC nor GCR possessed ROS scavenging activity *in vitro*. Increased amount of ROS can be generated by an imbalance of antioxidant enzymes and activation of the oxidase system. Membrane-bound nicotinamide adenine dinucleotide phosphate (NADPH) oxidase (Nox) is known to be a neurotoxin-related oxidase enzyme system [64], [65], and enzymatic antioxidants include superoxide dismutase (SOD), glutathione peroxidase (GPx), thioredoxin reductase (TPx) and catalase [66]. Therefore, it is likely that LPC and GCR might induce the imbalance by inhibiting oxidase activity directly or neurotoxin-induced activation of oxidase system. Furthermore, we can't exclude the possibility that LPC and GCR could induce the expression or activation of antioxidant enzymes.

**Figure 9:**
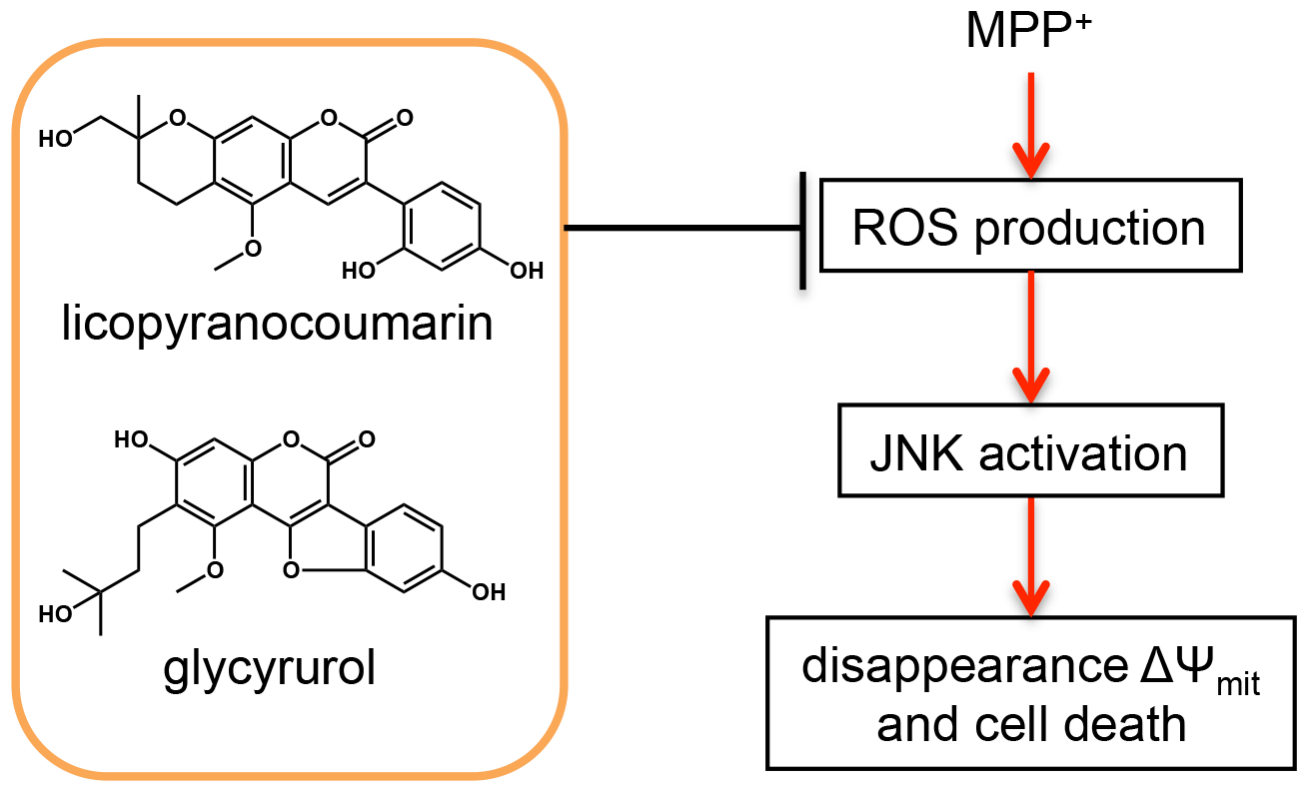
Suggested model for neuroprotection of licopyranocoumarin and glycyrurol against MPP^+^-induced toxicity in PC12D cells. Both licopyranocoumarin and glycyrurol exert neuroprotective effects against MPP^+^-induced toxicity via suppression of ROS generation and of JNK activation.

In summary, we identified *choi-joki-to* and *daio-kanzo-to* as neuroprotective herbal medicines, and both LPC and GCR were identified as neuroprotective substances from *Glycyrrhiza* contained in *choi-joki-to* and *daio-kanzo-to*. LPC or GCR exert their neuroprotective effects by inhibiting MPP^+^-induced ROS production and thus limiting JNK activation, and causing a subsequent decrease in ΔΨ_mit_. Our proposed mechanism is illustrated in Figure 9. Further studies are required to elucidate the molecular mechanisms for the suppression of ROS generation by LPC and GCR in PC12D cells. Our findings enliven the prospect of using LPC, GCR, *choi-joki-to* and *daio-kanzo-to* as effective and safe natural therapeutic agents in PD; *in vivo* trials in MPTP animal models are needed.

## Materials and Methods

### Reagents

MPP^+^, Rotenone, linoleic acid, 2,2-Diphenyl-1-pocrylhydrazyl (DPPH), SP600125 and mouse monoclonal anti-β-actin antibodies were purchased from Sigma Chemical Co. (St. Louis, MO). Taxol, cisplatin, JC-1 and pyridinium iodide were purchased from Wako Pure Chemical Industries, Ltd. (Osaka, Japan). Nerve growth factors, CM-H_2_DCFDA, and β-carotene standard were purchased from Alomone Labs (Jerusalem, Israel), Life Technologies (Carlsbad, CA) and Kanto Chemical Co. (Tokyo, Japan), respectively. Rabbit polyclonal anti-JNK antibody and rabbit monoclonal anti-phospho-JNK antibody were purchased from Santa Cruz Biotechnology (Santa Cruz, CA) and Cell Signaling (Beverly, MA), respectively. Horseradish peroxidase-conjugated anti-mouse and anti-rabbit IgG used as a secondary antibodies were from GE Healthcare (Little Chalfont, UK).

### Cell cultures

PC12D was identified a new subline of PC12 pheochromocytoma cells (PC12D cells) in which neurites are extended within 24 h in response to cAMP-enhancing reagents as well as in response to nerve growth factor (NGF) [30]. PC12D cells were cultured in Dulbecco's modified Eagle medium supplemented with 5% (v/v) inactivated fetal bovine serum, 10% (v/v) inactivated horse serum, 100 U/mL penicillin G, 0.6 mg/mL L-glutamine, and 0.1 mg/mL kanamycin at 37°C with 5% CO_2_. PC12D cells were differentiated by 100 ng/mL NGF treatment for 48 h.

### Cell viability assays

For the trypan blue dye exclusion assay, differentiated PC12D cells were cultured in 48-well dishes. Drug-treated or untreated cells were stained with trypan blue (Sigma Chemical Co.), and the ratio of viable cells was determined using a hemocytometer. Cell viability (%) means the ratio of the number of trypan blue-impermeable cells to total cell count. IC_50_ values were calculated by linear regression analysis from the inhibition of MPP^+^-induced cell death at different concentrations of the drug.

### Cell cycle analysis

To examine apoptosis, differentiated PC12D cells were harvested after drug treatment. The cells were washed with PBS and fixed with 70% ethanol at 4°C for more than 1 h. The cells were then stained with propidium iodide (PI) solution according to a previously reported protocol [67]. The labeled nuclei were subjected to flow cytometry (FCM, Beckman-Coulter, Miami, FL).

### Measurements of mitochondrial membrane potential

Changes in mitochondrial membrane potentials were assessed JC-1 (5,5′,6,6′-tetrachloro-1,1′,3,3′-tetraehylbenzimidazolylcarbocyanineiodide) (Wako) was used according to the manufacturer's protocol. Briefly, treated cells were collected by pipetting and removing medium. Next, the cells were incubated in medium containing 2.5 µg/ml JC-1 for 20 min at 37°C. Cells were then washed with PBS. JC-1 fluorescence was measured by a flow cytometer.

### Measurement of intracellular ROS

Intracellular ROS production was measured using CM-H_2_DCFDA. The cells were plated at a density of 12×10^4^ cells per 12-well dish. The cells were treated with MPP^+^ and test compounds for 12 h, and then trypsinized and collected. After the cells were washed with PBS, incubated with 2.5 µM CM-H_2_DCFDA in HBSS at 37°C for 30 min, and then washed again with PBS three times. The relative levels of fluorescence were quantified by using a flow cytometer.

### β-carotene bleaching assay

This assay was carried out according to the β-carotene bleaching method [68]. A mixture of β-carotene and linoleic acid was prepared by adding a mixture of 0.3 mg of β-carotene in 3 mL chloroform, 40 mg linoleic acid and 400 mg Tween 20. Chloroform was removed and 100 mL of distilled water was added to form an emulsion with continuous shaking. Aliquots (0.1 mL) of the β-carotene/linoleic acid emulsion were mixed with 1 µL of sample solution and incubated in a water bath at 50°C. The oxidation of the emulsion was monitored spectrophotometrically by measuring absorbance at 470 nm over a 60-min period. Control samples contained 1 µL of methanol. Antioxidant activity is expressed as percent inhibition relative to control after 60 min incubation using the following equation:

AA(%) = 100(DR_c_ – DR_s_)/DR_c_,

where AA = antioxidant activity; DR_c_ = degradation rate of the control = [ln(*a/b*)/60]; DR_s_ = degradation rate in presence of the sample = [ln(*a/b*)/60]; *a* = absorbance at time 0; *b* = absorbance at 60 min.

### DPPH radical scavenging assay

The DPPH radical scavenging effect of test compounds was determined according to the previously described method [68]. The reaction mixtures contained 100 µL ethanol, 125 µM DPPH, and test compounds. After 2 min of incubation at room temperature, the absorbance was recorded at 517 nm.

### Extraction and isolation of licopyranocoumarin and glycyrurol from *Glycyrrhiza*

Compounds were extracted from dried and pulverized *Glycyrrhiza* (50 g) with 90% EtOH, then filtrated and concentrated *in vacuo*. This suspension was adjusted to pH 7.0, followed by extraction with EtOAc (5 L) twice; the organic layer was concentrated to yield residue (3.76 g). The EtOAc extract was fractionated by centrifugal partition chromatography (CPC) with CHCl_3_:MeOH:H_2_O (5∶6∶4). The obtained crude active extract was applied on Sephadex LH20 column chromatography (Sephadex LH-20, 70 µM; GE Healthcare, NJ, USA), and eluted with MeOH. The active fraction (250.6 mg) was further purified by preparative octadecyl silyl (ODS) HPLC (YMC-Pack ODS-AQ, YMC Co. Ltd., Japan) with 40% aqueous CH_3_CN to give pure licopyranocoumarin (10.8 mg) and glycyrurol (4 mg), respectively.

### Western blotting

Cells were lysed in RIPA buffer (25 mM HEPES (pH 7.2), 1.5% Triton X-100 (Wako), 1% sodium deoxycholate (Wako), 0.1% SDS, 0.5 M NaCl (Wako), 5 mM EDTA, 50 mM NaF (Sigma), 0.1 mM sodium vanadate (Sigma) and 1 mM phenylmethylsulfonyl fluoride (PMSF) with sonication. The lysates were centrifuged at 13,000 rpm for 15 min to yield the soluble cell lysates. For immunoblotting, cell lysates were subjected to SDS-polyacrylamide gel electrophoresis. Proteins were transferred onto a polyvinylidene fluoride membrane (Millipore) by electroblotting and then incubated with appropriate antibodies. Immune complexes were detected with an Immobilon Western kit (Millipore), and luminescence was detected with a LAS-1000 mini (Fujifilm Co., Tokyo, Japan).

### Statistical analysis

All statistical analyses in bar plots were performed with a two-tailed paired Student's *t*-test.

## Supporting Information

Figure S1 **The correlation between contents of** ***Glycyrrhiza*** **and neuroprotective activity in herbal medicines.**
(TIF)

Figure S2 **Toxicity of EtOAc extract of** ***Glycyrrhiza*** **.** NGF-differentiated PC12D cells were treated with various concentrations of EtOAc extract of *Glycyrrhiza* for 48 h. Cell viability was evaluated by trypan blue dye exclusion assay.
(TIF)

Figure S3 **Licopyranocoumarin and glycyrurol preferentially showed cytoprotective effects in neuronal cells.** HeLa cells or NGF-differentiated PC12D cells were treated with various concentrations of licopyranocoumarin (LPC) or glycyrurol (GCR) in the presence of 0.3 µM Rotenone for 48 h. Cell viability was evaluated by trypan blue dye exclusion assay.
(TIF)
