# Supplementary figures and images for: Identification of Licopyranocoumarin and Glycyrurol from Herbal Medicines as Neuroprotective Compounds for Parkinson's Disease
Source: PLoS One. 2014 Jun 24;9(6):e100395. doi: 10.1371/journal.pone.0100395 (PMC4069009; doi:10.1371/journal.pone.0100395)

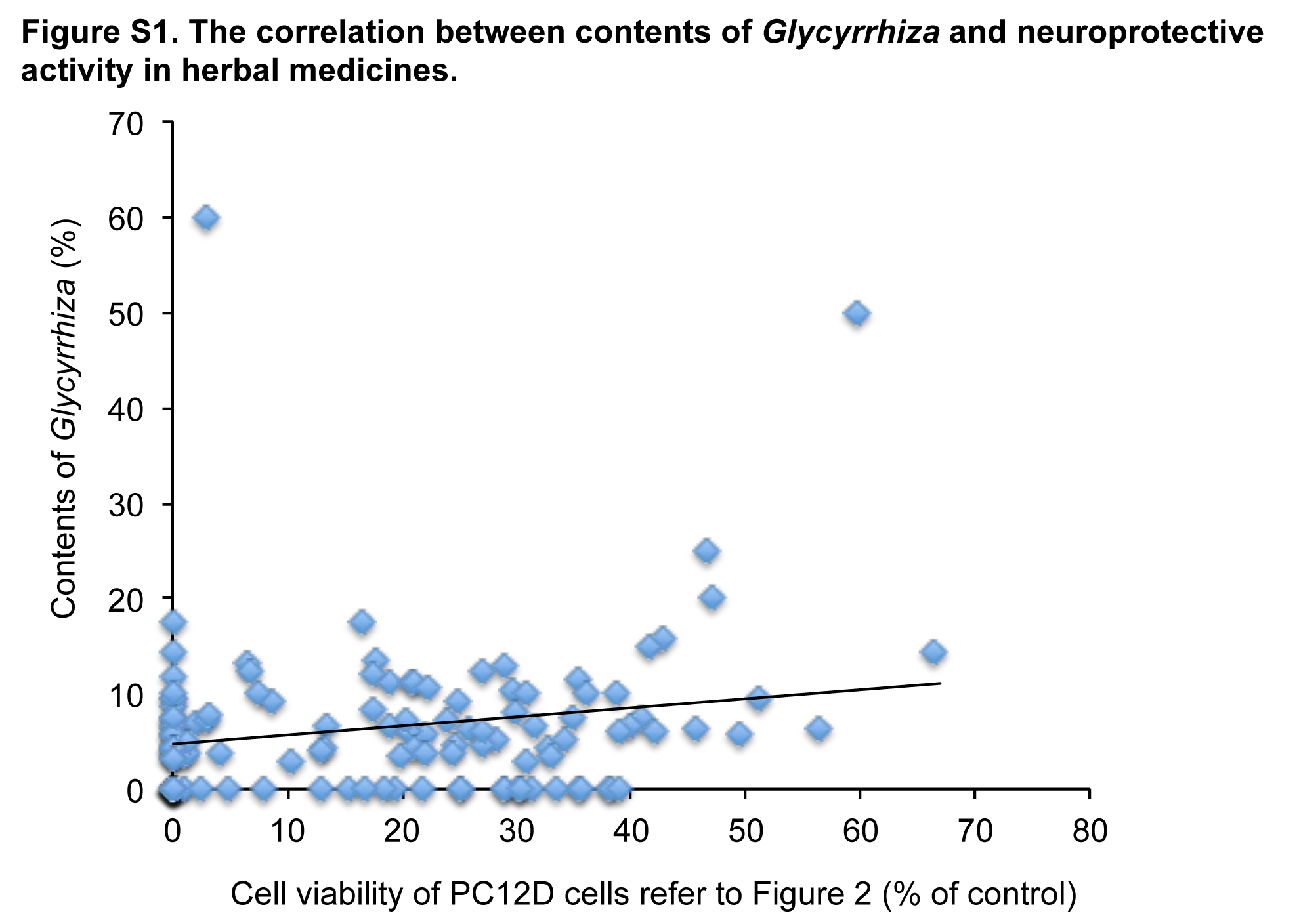

Supplement: Figure S1 — The correlation between contents of Glycyrrhiza and neuroprotective activity in herbal medicines. (TIF) [file pone.0100395.s001.tif]

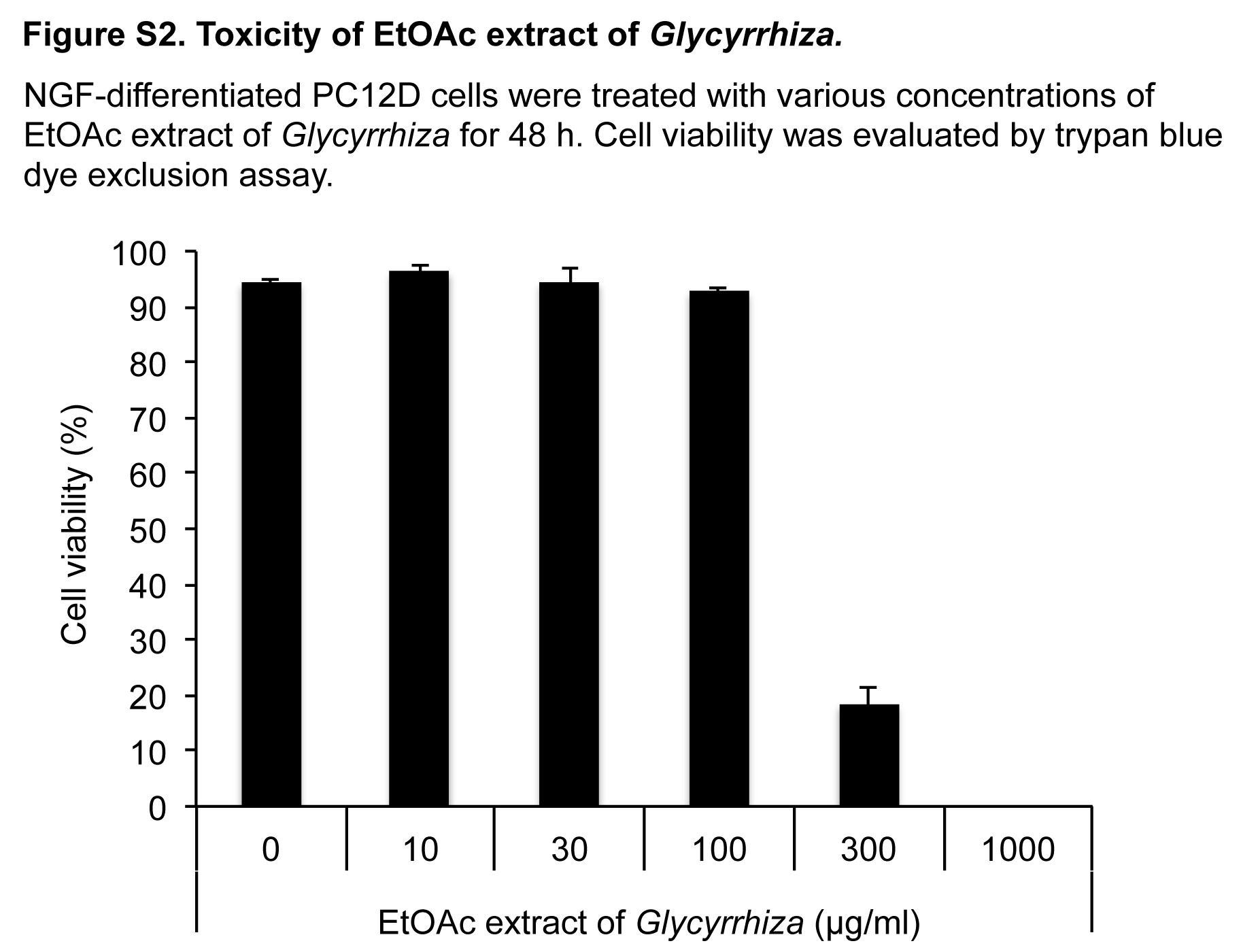

Supplement: Figure S2 — Toxicity of EtOAc extract of Glycyrrhiza . NGF-differentiated PC12D cells were treated with various concentrations of EtOAc extract of Glycyrrhiza for 48 h. Cell viability was evaluated by trypan blue dye exclusion assay. (TIF) [file pone.0100395.s002.tif]

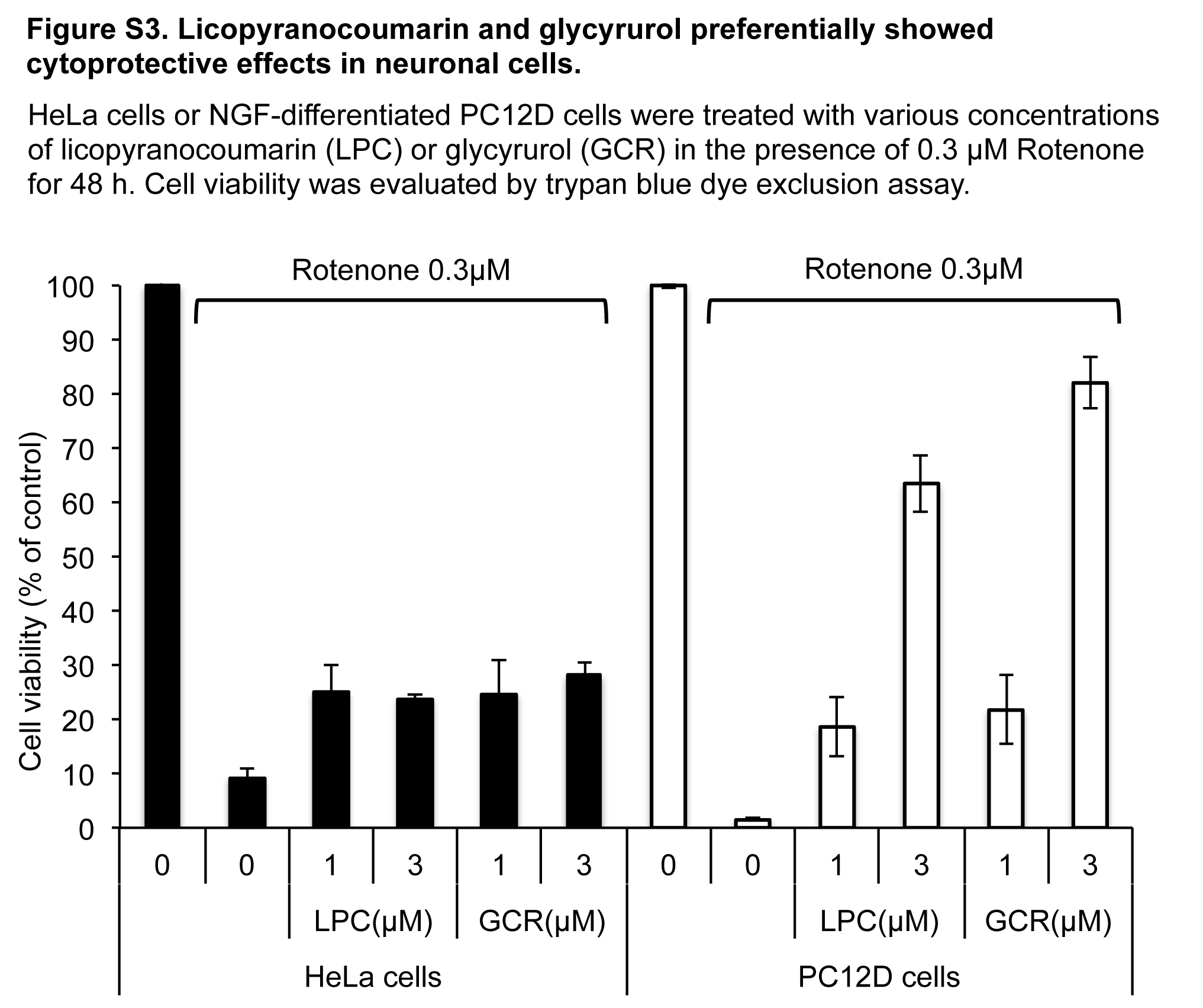

Supplement: Figure S3 — Licopyranocoumarin and glycyrurol preferentially showed cytoprotective effects in neuronal cells. HeLa cells or NGF-differentiated PC12D cells were treated with various concentrations of licopyranocoumarin (LPC) or glycyrurol (GCR) in the presence of 0.3 µM Rotenone for 48 h. Cell viability was evaluated by trypan blue dye exclusion assay. (TIF) [file pone.0100395.s003.tif]
